# Impact of levonorgestrel-releasing intrauterine device on amenorrhea rates after endometrial resection or ablation in women with abnormal uterine bleeding

**DOI:** 10.3389/fmed.2026.1662668

**Published:** 2026-04-28

**Authors:** Jiarui Qin, Xin Yang, Yinlei Liu, Cong Wan, Yanli Zheng

**Affiliations:** Department of Obstetrics and Gynecology, Nantong First People’s Hospital, Southeast University, Nantong, Jiangsu, China

**Keywords:** abnormal uterine bleeding, amenorrhea, endometrial ablation, endometrial resection, hysterectomy, levonorgestrel-releasing intrauterine device

## Abstract

**Objective:**

To evaluate the impact of adjunctive levonorgestrel-releasing intrauterine device (LNG-IUD) placement on amenorrhea rates and hysterectomy risk following endometrial resection or ablation in women with abnormal uterine bleeding (AUB).

**Methods:**

A retrospective cohort study was conducted involving 385 premenopausal women undergoing surgery for AUB. Patients were divided into surgery-only (*n* = 238) and surgery + LNG-IUD (*n* = 147) groups. Amenorrhea and hysterectomy rates were assessed at 12 months postoperatively. Multivariate logistic regression analyses were performed to examine the association of adjunctive LNG-IUD use with the risks of amenorrhea and hysterectomy, adjusting for potential confounders.

**Results:**

The surgery + LNG-IUD group demonstrated a significantly higher amenorrhea rate compared to the surgery-only group (61.2% vs. 24.4%, *p* < 0.01) and a lower hysterectomy rate (10.2% vs. 20.2%, *p* < 0.01). Multivariate logistic regression showed that adjunctive LNG-IUD use independently increased the odds of amenorrhea (adjusted OR = 4.89, 95% CI: 3.07–7.78, *p* < 0.01) and decreased the risk of hysterectomy (adjusted OR = 0.45, 95% CI: 0.24–0.85, *p* = 0.01).

**Conclusion:**

Adjunctive LNG-IUD placement significantly improves amenorrhea rates and reduces the risk of hysterectomy after endometrial surgery in women with AUB.

## Introduction

Abnormal uterine bleeding (AUB) is a common gynecologic complaint, affecting up to 30% of reproductive-aged women globally and accounting for nearly one-third of outpatient gynecology visits (1, 2). AUB significantly compromises quality of life, contributes to iron-deficiency anemia, and imposes a substantial healthcare burden (3, 4). In the United States alone, the estimated annual direct costs associated with AUB range from $1 to $1.55 billion, with indirect costs reaching $12 to $36 billion, largely due to surgical interventions and productivity loss (5).

Medical therapy is generally considered the first-line treatment, particularly for women desiring fertility preservation or those who wish to avoid surgical risks (6). Common pharmacologic options include combined oral contraceptives, oral progestins, tranexamic acid, and the levonorgestrel-releasing intrauterine device (LNG-IUD) (7). Among these, the LNG-IUD has shown the most consistent efficacy, achieving up to 80–90% reduction in menstrual blood loss and amenorrhea rates of 20–40% at 12 months (8, 9).

For patients who fail to respond to medical treatment or seek more durable symptom control, surgical options such as transcervical endometrial resection (TCRE) and radiofrequency endometrial ablation are frequently employed (10). These minimally invasive, uterus-preserving procedures offer lower morbidity compared to hysterectomy, which remains the most definitive but invasive option and is associated with higher rates of complications, infection, and prolonged recovery (10). Nonetheless, recurrence remains a challenge: up to 20–25% of women undergoing endometrial resection or ablation may require reintervention or hysterectomy within 3–5 years due to persistent or recurrent bleeding or pelvic pain (11, 12).

Recent studies have suggested that adjunctive placement of an LNG-IUD following endometrial resection or ablation may enhance clinical outcomes by increasing amenorrhea rates and reducing the likelihood of treatment failure or subsequent hysterectomy (13–15). However, robust evidence supporting this combination approach remains limited, particularly among Asian populations. Therefore, the present study aimed to evaluate the impact of LNG-IUD placement on amenorrhea outcomes in Chinese women undergoing endometrial resection or ablation for AUB.

## Methods

### Study design and population

This retrospective cohort study was conducted at Nantong First People’s Hospital, a tertiary care center in Nantong, China. We included women who underwent TCRE or endometrial ablation for AUB between January 2018 and December 2023. Patients were categorized into two groups according to whether they received postoperative levonorgestrel-releasing intrauterine device (LNG-IUD) placement and were followed for 12 months to assess clinical outcomes.

Participants were eligible for inclusion if they met all of the following criteria: (1) aged ≥35 years and premenopausal; (2) diagnosed with menorrhagia (ICD-10: N92.0), metrorrhagia (N92.1), or menometrorrhagia (N92.1B) based on clinical assessment and gynecologic history; (3) referred for and elected to undergo either TCRE or radiofrequency endometrial ablation; and (4) provided complete medical records and were available for at least 12 months of postoperative follow-up. Exclusion criteria included: (1) postmenopausal bleeding; (2) gynecological malignancies, including endometrial, cervical, or ovarian cancer (e.g., ICD-10: C53–C56, D07.0–D07.3); (3) structural uterine abnormalities such as submucosal fibroids >3 cm or uterine anomalies; (4) history of pelvic inflammatory disease within the past 6 months or active pelvic infection at the time of surgery; (5) known hypersensitivity to levonorgestrel or contraindications to IUD insertion; (6) prior hysterectomy or endometrial ablation/resection; and (7) use of systemic hormonal therapy during follow-up.

Eligible patients were divided into two groups based on postoperative management: those who received adjunctive placement of a levonorgestrel-releasing intrauterine device (LNG-IUD group) and those who did not (surgery-only group).

This study was approved by the Ethics Committee of Nantong First People’s Hospital (No. 2024022). All procedures were conducted according to the 1964 Declaration of Helsinki and its later amendments or comparable ethical standards. Informed consent was waived by the Ethics Committee of Nantong First People’s Hospital due to the retrospective nature.

### Surgical procedure and LNG-IUD placement

All patients underwent TCRE or radiofrequency endometrial ablation under general anesthesia, performed by experienced gynecologic surgeons following standard protocols. The choice of surgical technique was based on uterine characteristics, surgeon discretion, and equipment availability, rather than predefined study criteria. In the LNG-IUD group, a 52-mg levonorgestrel-releasing intrauterine device (Mirena^®^ or equivalent) was inserted immediately following completion of the surgical procedure, under direct visualization.

### Follow-up and outcome assessment

The primary outcome was the rate of amenorrhea at 12 months, defined as complete cessation of menstrual bleeding for at least 3 consecutive months. Secondary outcomes included the rate of hysterectomy during the observation period.

Demographic data, baseline clinical characteristics, and intraoperative details were collected from electronic medical records. Follow-up information was obtained through outpatient visits and standardized telephone interviews conducted by trained research staff blinded to the study hypothesis.

### Statistical analysis

Continuous variables were presented as means ± standard deviations and compared using the Student’s *t*-test or Mann–Whitney U test, as appropriate. Categorical variables were expressed as frequencies and percentages and compared using chi-square or Fisher’s exact tests.

To address potential selection bias and confounding in this retrospective study, we employed several strategies. First, we compared all baseline demographic and clinical characteristics between the two groups to identify any significant differences. Second, we performed multivariate logistic regression analyses to adjust for identified and potential confounders.

Multivariate logistic regression was performed to evaluate the independent association between LNG-IUD use and risks of amenorrhea and hysterectomy, after adjusting for potential confounding factors including age, body mass index (BMI), smoking, birth, cesarean section, other uterine surgeries, and PALM-COEIN classification.

A two-sided *p* < 0.05 was considered statistically significant. All analyses were performed using SPSS software (version 27.0; IBM Corp., Armonk, NY, United States).

## Results

A total of 385 women were included in the final analysis, with 238 in the surgery-only group and 147 in the surgery + LNG-IUD group. Baseline demographic and clinical characteristics are summarized in Table 1. There were no statistically significant differences between the two groups in terms of age, BMI, or smoking status (all *p* > 0.05). The distribution of preoperative referral diagnoses (menorrhagia, metrorrhagia, and menometrorrhagia) did not differ significantly between groups (*p* = 0.35). Other clinical variables, including birth, prior cesarean section, history of uterine surgeries, and PALM-COEIN classification, were also comparable across groups (all *p* > 0.05).

**Table 1:** Baseline demographic data for the participants included in the surgery + LNG-IUD and surrey groups.

Variables	Total	Surgery only	Surgery + LNG-IUD	*P*
*N*	385	238	147	
Age, years	45.59 ± 5.65	45.29 ± 5.94	46.08 ± 5.14	0.17
BMI, kg/m^2^	24.46 ± 1.47	24.53 ± 1.42	24.35 ± 1.53	0.23
Smoking, n (%)				0.12
No	340 (88.3%)	215 (90.3%)	125 (85.0%)	
Yes	45 (11.7%)	23 (9.7%)	22 (15.0%)	
Preoperative referral diagnosis, n (%)				0.35
Menorrhagia	213 (55.3%)	125 (52.5%)	88 (59.9%)	
Metrorrhagia	67 (17.4%)	45 (18.9%)	22 (15.0%)	
Menometrorrhagia	105 (27.3%)	68 (28.6%)	37 (25.2%)	
Birth, n (%)				0.42
No	26 (6.8%)	18 (7.6%)	8 (5.4%)	
Yes	359 (93.3%)	220 (92.4%)	139 (94.6%)	
Cesarean section, n (%)				0.54
No	300 (77.9%)	183 (76.9%)	117 (79.6%)	
Yes	85 (22.1%)	55 (23.1%)	30 (20.4%)	
Other uterine surgeries, n (%)				0.97
No	306 (79.5%)	189 (79.4%)	117 (79.6%)	
Yes	79 (20.5%)	49 (20.6%)	30 (20.4%)	
PALM-COEIN Classification, n (%)				0.31
AUB-P (Polyp)	38 (9.9%)	25 (10.5%)	13 (8.8%)	
AUB-L (leiomyoma)	62 (16.1%)	42 (17.6%)	20 (13.6%)	
AUB-A (adenomyosis)		28 (7.3%)	13 (5.5%)	15 (10.2%)
AUB-M (hyperplasia)	50 (13.0%)	28 (11.8%)	22 (15.0%)	
AUB-E (endometrial)	207 (53.8%)	130 (54.6%)	77 (52.4%)	

**Figure 1:**
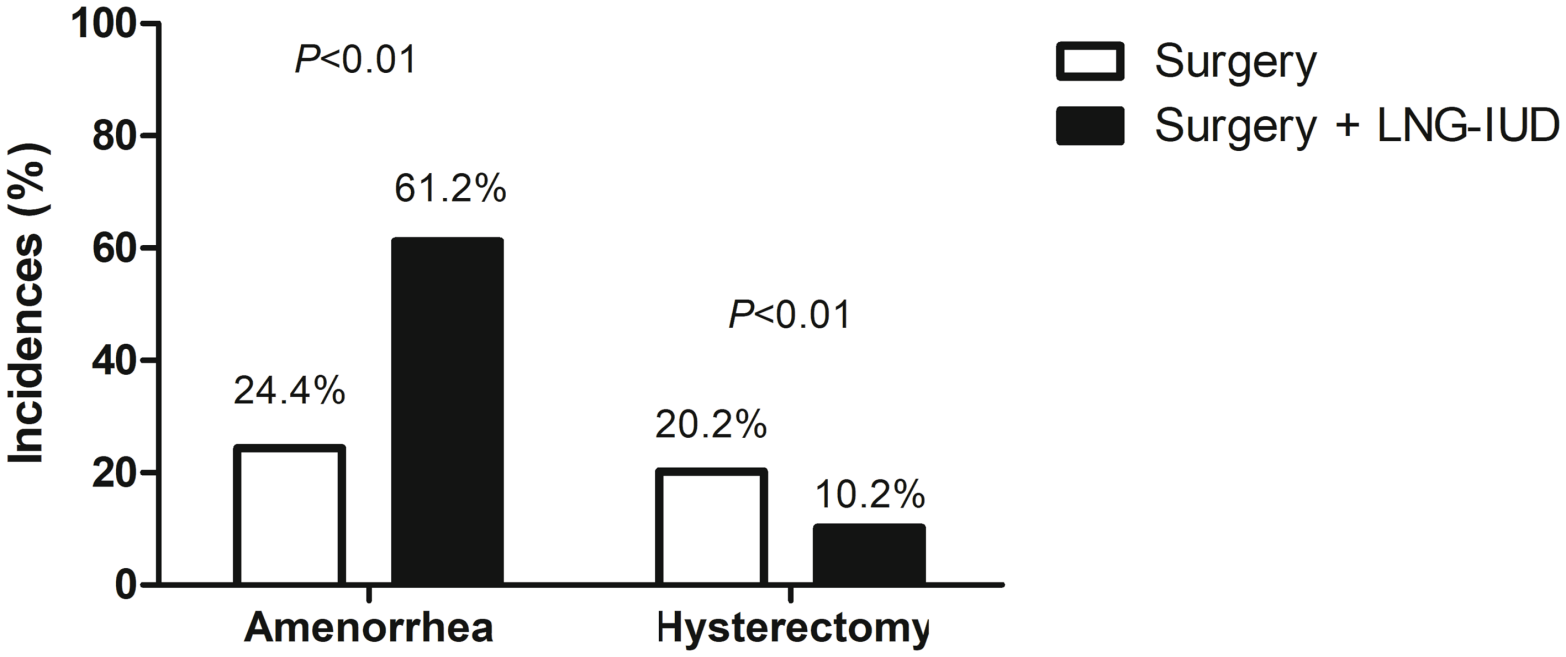
Comparison of amenorrhea and hysterectomy rates between the surgery-only group and the surgery + LNG-IUD group.

At 12-month follow-up, the rate of amenorrhea was significantly higher in the surgery + LNG-IUD group compared to the surgery-only group (61.2% vs. 24.4%, *p* < 0.01) (Figure 1). Univariate logistic regression revealed that adjunctive LNG-IUD use was associated with a markedly increased likelihood of amenorrhea (OR = 4.90, 95% CI: 3.14–7.64, *p* < 0.01). After adjusting for potential confounding factors in multivariate logistic regression, LNG-IUD placement remained an independent predictor of amenorrhea (OR = 4.89, 95% CI: 3.07–7.78, *p* < 0.01) (Table 2). In addition to LNG-IUD use, older age was independently associated with higher odds of amenorrhea (OR = 1.06, 95% CI: 1.02–1.11, *p* < 0.01). Other covariates, including BMI, smoking, preoperative diagnosis, birth, cesarean section, prior uterine surgeries, and PALM-COEIN classification, were not significantly associated with amenorrhea (all *p* > 0.05) (Table 2).

**Table 2:** Univariate and multivariate logistic regression analyses to explore the risk factors of amenorrhea.

Variables	Univariate regression			Multivariate regression		
	OR	95% CI	*P*	OR	95% CI	*P*
Group						
Surgery only	Reference			Reference		
Surgery + LNG-IUD	4.90	3.14–7.64	<0.01	4.89	3.07–7.78	<0.01
Age	1.06	1.02–1.10	<0.01	1.06	1.02–1.11	<0.01
BMI	0.87	0.76–1.00	0.05	0.87	0.74–1.02	0.08
Smoking						
No	Reference			Reference		
Yes	1.63	0.87–3.03	0.13	1.66	0.82–3.35	0.16
Birth						
No	Reference			Reference		
Yes	1.76	0.72–4.28	0.22	1.64	0.62–4.34	0.32
Cesarean section						
No	Reference			Reference		
Yes	1.16	0.71–1.89	0.56	1.38	0.79–2.40	0.26
Other uterine surgeries						
No	Reference			Reference		
Yes	0.98	0.59–1.62	0.92	0.98	0.56–1.71	0.93
PALM-COEIN classification						
AUB-P (polyp)	Reference			Reference		
AUB-L (leiomyoma)	1.90	0.82–4.44	0.14	2.39	0.93–6.11	0.07
AUB-A (adenomyosis)	2.89	1.05–7.96	0.04	2.49	0.81–7.68	0.11
AUB-M (hyperplasia)	1.44	0.60–3.51	0.42	1.19	0.44–3.21	0.73
AUB-E (endometrial)	1.13	0.54–2.38	0.75	1.12	0.49–2.55	0.80

During the 12-month follow-up, hysterectomy was performed in 63 patients, including 48 (20.2%) in the surgery-only group and 15 (10.2%) in the surgery + LNG-IUD group (*p* < 0.01) (Figure 1). Among those in the surgery + LNG-IUD group who underwent hysterectomy, the median duration of device use before surgery was 10 months (IQR, 7–12). The primary reasons for hysterectomy were persistent heavy bleeding despite LNG-IUD treatment (60.0%), unresolved pelvic pain (33.3%), and spontaneous expulsion of the device (6.7%). In the surgery-only group, hysterectomy was mainly attributed to persistent or recurrent heavy bleeding (62.5%) and refractory pelvic pain (37.5%). Univariate logistic regression demonstrated that adjunctive LNG-IUD use significantly reduced the odds of hysterectomy (OR = 0.45, 95% CI: 0.24–0.84, *p* = 0.01), and this association remained significant after adjustment for confounding variables (OR = 0.45, 95% CI: 0.24–0.85, *p* = 0.01) (Table 3).

**Table 3:** Univariate and multivariate logistic regression analyses to explore the risk factors of hysterectomy.

Variables	Univariate regression			Multivariate regression		
	OR	95% CI	*P*	OR	95% CI	*P*
Group						
Surgery only	Reference			Reference		
Surgery + LNG-IUD	0.45	0.24–0.84	0.01	0.45	0.24–0.85	0.01
Age	0.96	0.91–1.01	0.08	0.97	0.92–1.01	0.15
BMI	1.03	0.86–1.24	0.77	1.03	0.85–1.25	0.73
Smoking						
No	Reference			Reference		
Yes	1.55	0.72–3.31	0.26	1.57	0.70–3.51	0.27
Birth						
No	Reference			Reference		
Yes	0.50	0.20–1.25	0.14	0.54	0.21–1.40	0.21
Cesarean section						
No	Reference			Reference		
Yes	0.62	0.30–1.28	0.20	0.62	0.29–1.31	0.21
Other uterine surgeries						
No	Reference			Reference		
Yes	1.26	0.66–2.39	0.48	1.19	0.61–2.32	0.61
PALM-COEIN classification						
AUB-P (polyp)	Reference			Reference		
AUB-L (leiomyoma)	0.48	0.16–1.45	0.19	0.46	0.15–1.43	0.18
AUB-A (adenomyosis)	0.29	0.06–1.48	0.14	0.30	0.06–1.60	0.16
AUB-M (hyperplasia)	0.71	0.24–2.12	0.54	0.75	0.24–2.32	0.62
AUB-E (endometrial)	0.84	0.36–1.98	0.70	0.83	0.34–2.03	0.68

When stratified by surgical technique, adjunctive LNG-IUD use was consistently associated with higher amenorrhea rates and lower hysterectomy rates compared with surgery alone in both the TCRE and radiofrequency ablation subgroups (Table 4). In the TCRE subgroup, adjunctive LNG-IUD use was independently associated with a markedly increased likelihood of amenorrhea (adjusted OR = 6.84, 95% CI 3.60–13.00; *p* < 0.01) and a significantly reduced risk of hysterectomy (adjusted OR = 4.02, 95% CI 1.89–8.57; *p* < 0.01) compared with TCRE alone. In the radiofrequency ablation subgroup, adjunctive LNG-IUD use was also associated with a higher likelihood of amenorrhea (adjusted OR = 0.41, 95% CI 0.17–0.98; *p* = 0.04) and a significantly lower risk of hysterectomy (adjusted OR = 0.55, 95% CI 0.21–0.85; *p* = 0.02).

**Table 4:** Multivariate logistic regression analyses to explore the association of LNG-IUD with risks of amenorrhea and hysterectomy stratified by surgical techniques.

Variables	No. of events			Multivariate regression		
	Surgery only	Surgery + LNG-IUD	*P*	OR	95% CI	*P*
TCRE	145	87				
Amenorrhea	38 (26.2%)	60 (69.0%)	<0.01	6.84	3.60–13.00	<0.01
Hysterectomy	28 (19.3%)	8 (9.2%)	0.04	4.02	1.89–8.57	<0.01
Ablation	93	60		0.97	0.92–1.01	0.15
Amenorrhea	20 (21.5%)	30 (50.0%)	<0.01	0.41	0.17–0.98	0.04
Hysterectomy	20 (21.5%)	7 (50.0%)	0.12	0.55	0.21–0.85	0.02

## Discussion

This retrospective cohort study demonstrated that adjunctive placement of an LNG-IUD at the time of endometrial resection or ablation significantly improved amenorrhea outcomes and reduced hysterectomy rates in women with AUB. Specifically, the LNG-IUD group had more than a four-fold increase in the odds of achieving amenorrhea at 12 months compared to the surgery-only group, even after adjusting for age, BMI, and clinical history. Additionally, the use of LNG-IUD was associated with a 55% reduction in the odds of hysterectomy during the follow-up period.

Our findings are consistent with previous studies demonstrating the benefit of adjunctive LNG-IUD use following endometrial resection or ablation in improving amenorrhea rates and reducing hysterectomy risk. For example, a prospective cohort study by Straarup et al. (16) found that the 12-month amenorrhea rate increased from 11% with TCRE alone to 58% when combined with LNG-IUD (OR = 24.71, 95% CI: 2.32–262.94). Similarly, Darre Haahr et al. (14) reported an amenorrhea rate of 59% in the TCRE + LNG-IUD group (OR = 2.56, 95% CI: 1.46–4.49, *p* < 0.01), along with significantly lower adjusted odds of hysterectomy (OR = 0.35, 95% CI: 0.13–0.97). Compared to these Western studies, our study confirms the advantages of this combined approach in a Chinese population using real-world clinical data. The magnitude of benefit observed in our cohort was comparable to that reported previously, supporting the generalizability of this treatment strategy across different populations.

Several mechanisms may underlie the enhanced therapeutic efficacy observed with the combination of surgery and LNG-IUD. The LNG-IUD delivers a high local concentration of levonorgestrel directly to the endometrial lining, leading to profound suppression of endometrial activity, glandular atrophy, and reduced angiogenesis (17). This hormonal environment likely complements the mechanical removal or destruction of endometrial tissue achieved through resection or ablation, thereby promoting sustained amenorrhea. In addition, the presence of the LNG-IUD may inhibit endometrial regrowth and re-epithelialization, processes that are commonly implicated in treatment failure or symptom recurrence after surgical intervention alone (18). Moreover, levonorgestrel’s anti-inflammatory and antiproliferative properties may help resolve subclinical endometrial inflammation, further reducing the risk of persistent or recurrent bleeding (19).

It should be acknowledged that, without a comparison group receiving LNG-IUD alone, our study cannot definitively distinguish true pharmacologic–surgical synergy from additive hormonal effects or determine the independent contribution of each intervention. Nonetheless, the observed amenorrhea rate in the combination group (61.2%) substantially exceeds typical rates for LNG-IUD monotherapy (20–40%) (8, 9), suggesting additive or complementary benefits. This enhanced efficacy may reflect the combined effect of mechanical endometrial removal or ablation with local progestin-induced endometrial suppression, glandular atrophy, and inhibition of regrowth, which together promote sustained amenorrhea. Furthermore, inclusion of hysterectomy as a clinically meaningful secondary endpoint indicates that improved bleeding control was not merely symptomatic masking, but translated into a reduced need for subsequent definitive surgery. These findings support the effectiveness of combining endometrial resection or ablation with LNG-IUD placement in real-world practice, although longer-term and randomized studies are warranted to confirm sustained benefit and clarify mechanistic interactions.

This study has several strengths, including a well-defined patient population, standardized surgical technique, and adjustment for multiple confounders. However, some limitations should be acknowledged. First, the retrospective and non-randomized design of this study may introduce selection bias, as the decision to place an LNG-IUD could reflect patient preferences or physician practice patterns rather than random allocation. Although baseline characteristics were comparable between groups and multivariate analyses were performed to adjust for measured confounders, the influence of unmeasured factors cannot be completely excluded. For instance, several clinically relevant factors—including endometrial thickness, use of medical therapy prior to surgery, fertility intentions, and the specific indications guiding the choice of adjunctive LNG-IUD placement—were not uniformly available in this retrospective dataset and therefore could not be analyzed. These factors may have influenced both treatment selection and outcomes. Second, the inclusion of two different endometrial surgical techniques may have introduced procedural heterogeneity. Although subgroup analyses stratified by surgical technique were performed, these analyses were exploratory in nature and limited by sample size, precluding definitive subgroup-specific conclusions. Third, the amenorrhea outcome was based on patient self-report, which may introduce recall bias. Finally, the follow-up period was limited to 12 months; longer-term data are needed to assess sustained efficacy and hysterectomy-free survival.

## Conclusion

In conclusion, our findings support the adjunctive use of LNG-IUD in women undergoing endometrial resection or ablation for AUB. This combined approach significantly increases amenorrhea rates and reduces the likelihood of hysterectomy. Future prospective randomized controlled trials with longer follow-up, standardized outcome measures, and quality-of-life assessments are warranted to further validate these findings and establish evidence-based guidelines for this combined uterus-sparing treatment strategy.

## Data Availability

The raw data supporting the conclusions of this article will be made available by the authors, without undue reservation.
